# Two cases of pelvic lymphocele after prostatectomy and dissection of obturator lymph nodes successfully treated by interventional radiology

**DOI:** 10.1002/iju5.12337

**Published:** 2021-06-28

**Authors:** Yuki Oda, Nobuo Ohyama, Masahiro Hashimura, Shinsaku Maeda, Shunta Hori, Kiyohide Fujimoto

**Affiliations:** ^1^ Department of Urology Nara Prefecture Seiwa Medical Center Kitakatsuragi Japan; ^2^ Department of Radiology Nara Prefecture Seiwa Seiwa Medical Center Kitakatsuragi Japan; ^3^ Department of Urology Nara Medical University Kashihara Japan

**Keywords:** lymphangiography, *N*‐butyl‐cyanoacrylate, Refractory lymphocele

## Abstract

**Introduction:**

Postoperative refractory lymphocele is often difficult to treat. Recently, interventional radiology with *N*‐butyl‐cyanoacrylate has been used by urologists and radiologists to treat lymphocele. This modality is an effective treatment with fewer complications.

**Case presentation:**

Case 1. A 70‐year‐old man, who underwent retropubic radical prostatectomy and bilateral obturator lymph node dissection, developed postoperative lymphocele. Continuous drainage and multiple rounds of sclerotherapy to reduce lymphocele volume ended in failure. Subsequently, lymphangiography with lipiodol and *N*‐butyl‐cyanoacrylate was performed, and the lymphocele volume gradually decreased.

Case 2. A 75‐year‐old man underwent retropubic radical prostatectomy and bilateral obturator lymph node dissection. After surgery, the patient developed a high‐output lymphocele. The lymphocele volume decreased following lymphangiography with lipiodol.

**Conclusion:**

Interventional radiology using lipiodol and *N*‐butyl‐cyanoacrylate could provide a new standard treatment for refractory lymphocele.

**Abbreviations & Acronyms**
CT: computed tomography
DVT: deep vein thrombosis
IV: iliac vein
IVR: interventional radiology
LC: lymphocele
NBCA: *N*‐butyl‐cyanoacrylate
OLND: obturator lymph node dissection
PLND: pelvic lymph node dissection
RRP: retropubic radical prostatectomy
SLC: symptomatic lymphocele
VTE: venous thromboembolism

**Keynote message**

Generally, postoperative lymphoceles are treated with highly invasive surgical procedures. With the advancement of interventional radiology, lymphangiography using lipiodol and *N*‐butyl‐cyanoacrylate is a relatively less invasive procedure.

## Introduction

Most lymphoceles disappear asymptomatically without treatment. However, some patients require therapeutic interventions, including conservative treatment, percutaneous drainage, sclerotherapy, and surgery.^1^ Moreover, patients with refractory lymphocele may be difficult to treat and have prolonged hospitalizations.

Recently, IVR has received much attention in the treatment of postoperative lymphocele.^2^ Lymphangiography using lipiodol, and occasionally in combination with NBCA, has been cited in cases of refractory lymphocele.^3^, ^4^, ^5^, ^6^

In this report, we describe the effect of IVR in patients with refractory postoperative lymphocele.

## Case presentation

We report two cases of lymphocele after RRP and OLND. In both cases, we ligated peripheral sides and sealed rest of the sides using a sealing device at the process of OLND.

**Fig. 1:**
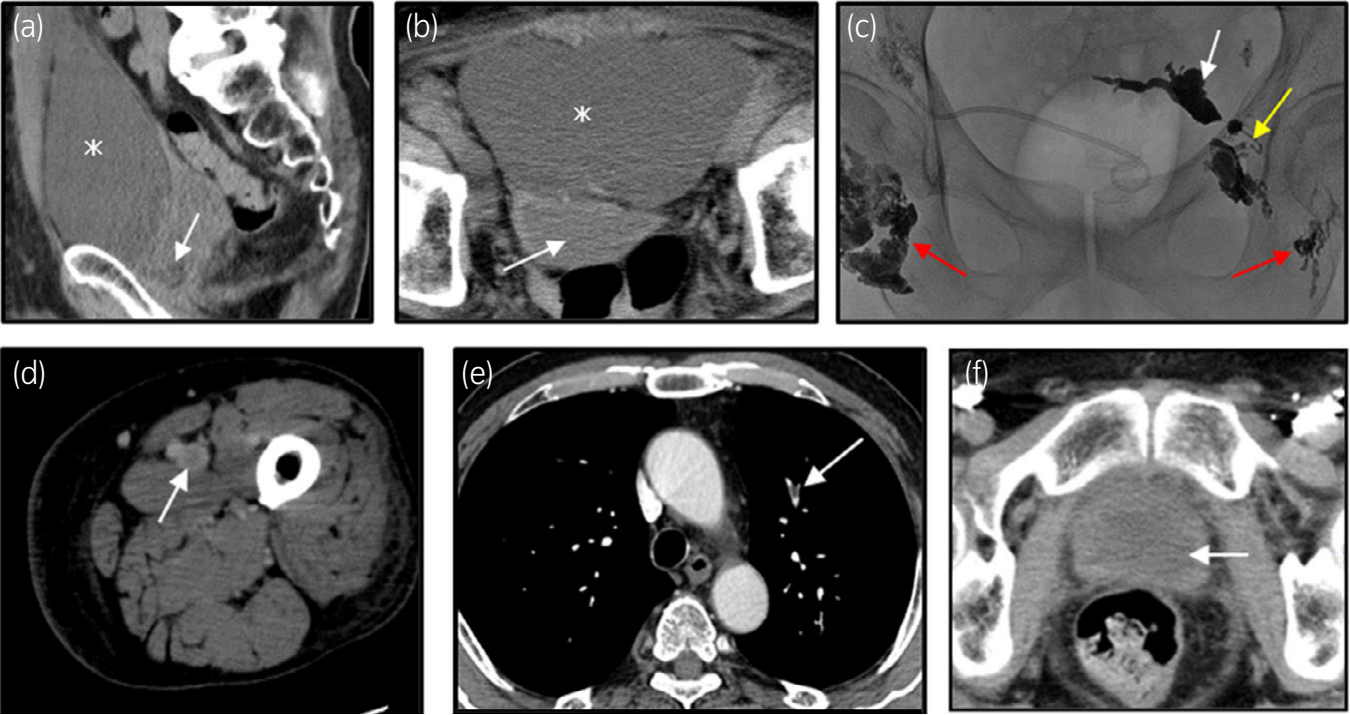
Representative images of Case 1 during treatment. Abdominal CT shows a large lymphocele in front of the bladder (a: sagittal image, b: axial image. The asterisk indicates the lymphocele; the white arrow indicates the bladder). The left lower extremity CT shows a deep vein thrombosis (c: axial image, white arrow). The pulmonary artery CT shows pulmonary vein thrombosis (d: axial image, white arrow). The representative lymphangiography image of Case 1: The left side of the lymphangiography shows lymphatic leakage in the upper stream of the lymphatic vessel (e: red arrows indicate the bilateral inguinal nodes; yellow arrow indicates the left lymphatic vessel; and white arrow indicates the lymphatic leakage site). Abdominal CT was performed 50 days after RRP. The lymphocele had completely disappeared (f: axial image, white arrow indicates the bladder).

Case 1 was a 70‐year‐old man with prostate adenocarcinoma of Gleason score 9. He underwent RRP and bilateral OLND. Twelve days after the surgery, he presented at our hospital with a complaint of abdominal swelling and left pedal edema. CT showed a large lymphocele in front of the bladder (Fig. 1a,b), DVT in the left femoral vein (Fig. 1c), and pulmonary embolisms in the bilateral peripheral pulmonary arteries (Fig. 1d). Anticoagulation therapy was started, and a 6.5 Fr pigtail catheter was placed into the pelvis to drain the lymphocele. Despite this, the lymphatic‐drainage volume was more than 500 mL/day. Therefore, we attempted sclerotherapy with minocycline 11 times, and with anhydrous ethanol two times. No adverse events associated with sclerotherapy, such as pelvic pain or fever, were detected. The lymphatic‐drainage volume decreased slightly to over 200 mL/day. Subsequently, lymphangiography was performed by a radiologist at our hospital to locate the lymphatic leak point (Fig. 1e). A 23G injection needle was used to puncture bilateral inguinal lymph nodes, and lipiodol was injected through the puncture sites. Leakage of lipiodol was detected in the left upper lymphatic vessel. The lipiodol was insufficient to embolize the area of lymphatic leakage. Therefore, NBCA, diluted 1:10 with lipiodol, was injected into the puncture sites to obstruct the leakage. Thus, the lymphatic leak point was successfully embolized, and the drainage catheter was removed. Twenty days after lymphangiography and lymphatic embolization using NBCA, the lymphocele disappeared with no evidence of recurrence (Fig.1f).

**Fig. 2:**
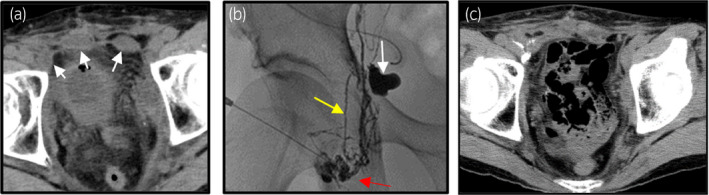
Representative images of Case 2 during treatment. Abdominal CT shows three sites of small lymphocele in front of the bladder (a: axial image, white arrows indicate the small lymphoceles). Representative lymphangiography image of Case 2. The right side of the lymphangiography shows lymphatic leakage at the upper stream of the right lymphatic vessel (b: red arrow indicates the right inguinal node; yellow arrow indicates the right lymphatic vessel; and white arrow indicates the lymphatic leakage site.). Abdominal CT taken 18 days after RRP (c: axial image). The lymphoceles have almost completely disappeared.

Case 2 was a 75‐year‐old man with a history of prostate adenocarcinoma of Gleason score 10. He was hospitalized in our department and underwent RRP and bilateral OLND. A drainage tube was inserted into the pelvic region, and a urethral catheter was placed. Five days after the surgery, cystography showed a leak from the vesicourethral anastomosis. Then another week was needed to close the leakage. After removal of a urethral catheter, the fluid volume from a pelvic drain continued to be over 150 mL/day, and lymphatic disruption was suspected. CT images showed three sites of small lymphoceles in front of the bladder (Fig. 2a). Sixteen days after RRP, lymphangiography was performed by a radiologist (Fig. 2b). The right inguinal lymph node was punctured using a 23G injection needle, and lipiodol was injected through the puncture site. The leakage of lipiodol was detected in the right upper lymphatic vessel, but it ceased shortly after the lipiodol injection. Eighteen days after RRP, the pelvic drain slipped out naturally, and pelvic CT demonstrated little evidence of lymphocele in the pelvis (Fig. 2c). Twenty‐one days after RRP, the patient was discharged from our hospital, and no recurrence was reported.

## Discussion

Lymphangiography with lipiodol causes hydrolysis and saponification of the adipose tissue surrounding the lymphocele, leading to a granulomatous reaction with fibrosis and subsequent lymphatic leak obstruction.^2^, ^6^, ^7^ In fact, recent studies have shown a 46‐89% success rate in obstructing lymphatic leaks by using lipiodol in lymphangiography.^2^, ^7^, ^8^

NBCA is used as an adhesive for skin sutures, vascular aneurysm treatment, and vascular embolization. Recently, an increasing number of cases has been reported in which NBCA was used for lymphatic embolization.^3^, ^4^, ^5^, ^6^ Lipiodol in combination with NBCA has an 80‐100% success rate in lymphatic embolization. Woo et al. reported that lymphatic embolization complications associated with the use of NBCA included infection and lower extremity lymphedema. However, the incidence rate of NBCA complications is only 0–20%. Moreover, none of these complications were reported to be severe (Table 1).^1^, ^3^, ^4^, ^5^, ^6^, ^8^, ^9^, ^10^, ^11^, ^12^, ^13^, ^14^, ^15^

**Table 1:** Previous reports of lymphatic embolization using lipiodol and NBCA

Authors	No. patients	Success	Complication	Ref
Ron et al	1	1 (100%)	0	1
Itou et al	1	1 (100%)	0	11
Ching et al	1	1 (100%)	0	12
Dinc et al	1	1 (100%)	0	13
Beak et al	5	4 (80%)	1 (20%)	14
Hur et al	16	15 (94%)	2 (12.5%)	8
Beak et al	21	20 (95.2%)	0	5
Srinivasa et al	1	1 (100%)	0	15
Hill et al	4	4 (100%)	0	6
Smolock et al	10	8 (80%)	0	3
Kayama et al	1	1 (100%)	0	16
Chu et al	9	9 (100%)	0	4
Kim et al	24	20 (83.3%)	2 (8.3%)	9

The success rate ranges from 80 to 100%. No severe complications were reported in using NBCA for lymphatic embolization.

The incidence of lymphocele in patients after RRP and PLND is up to 61%.^16^ Most of these patients are asymptomatic. However, some develop SLC mainly in the form of low‐extremity edema and VTE. In the worst case, an SLC patient with these complications may develop sepsis or pulmonary thromboembolism.^16^

**Fig. 3:**
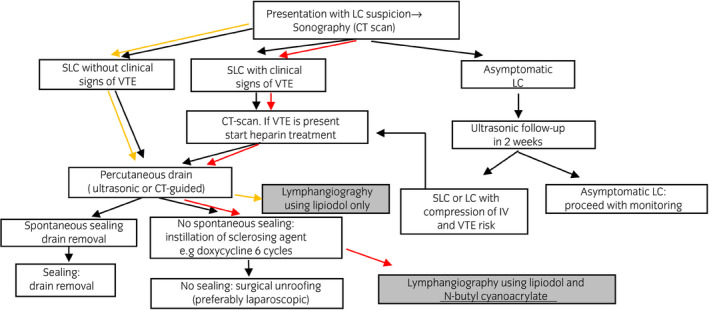
Tsaur et al proposed the algorithm of SLC treatment. Traditional SLC treatments are percutaneous drainage, sclerotherapy, and surgery (black arrows). Case 1 was a refractory SLC. Ordinarily, the patient would have undergone surgery to block lymphatic leakage. However, we performed IVR, lipiodol lymphangiography, and lymphatic embolization with NBCA. The lymphocele disappeared after treatment (red arrows). Case 2 was also a refractory SLC. Based on Case 1 experience, we selected lymphangiography with lipiodol before sclerotherapy (orange arrows). These two cases have demonstrated that refractory lymphocele patients should undergo lymphangiography using lipiodol and NBCA at an early stage of treatment.

Tsaur et al. demonstrated a treatment algorithm for SLC (Fig. 3).^16^ Traditionally, refractory lymphoceles are treated with sclerotherapy. When this fails, surgical intervention is commonly performed. However, surgical intervention is highly invasive, and is accompanied by complications such as adhesion and infection. Conversely, IVR is relatively safe. IVR could therefore be an appealing alternative to surgical intervention.

Lymphatic leakage in Case 1 did not stop, despite continuous drainage and 13 times of sclerotherapies. Although surgery would have been the next standard treatment, the radiologist performed IVR, lipiodol lymphangiography, and lymphatic embolization with NBCA instead (Fig. 3). This case demonstrates that lymphatic angiography may be a viable alternative to sclerotherapy for refractory lymphocele. Lymphatic angiography may be used to simultaneously locate the lymphatic leak point and embolize the leakage using lipiodol alone, or in combination with NBCA.

Based on our experience with Case 1, when continuous drainage in Case 2 failed to stop the lymphatic leak, lymphangiography with lipiodol was administered directly, without the intervention of sclerotherapy (Fig. 3). As a result, lymphatic leakage was successfully treated.

These two cases suggest that a refractory lymphocele patient should undergo lymphangiography using lipiodol and NBCA at an early stage of treatment, because IVR is minimally invasive for patients, and it requires shorter periods of hospitalization.

## Conclusion

Based on our two cases, when lymphatic leakage in a patient with postoperative lymphocele does not improve despite continuous drainage, lymphangiography should be considered as the next treatment. In some cases, concomitant lymphatic embolization with NBCA should be performed.

## Conflict of interest

The authors declare no conflict of interest.

## Approval of the research protocol by an Institutional Reviewer Board

Not applicable.

## Informed consent

All informed consent was obtained from the participants.

## Registry and the Registration No. of the study/trial

Not applicable.
